# The Development and Validation of a Revised Version of the Math Anxiety Scale for Young Children

**DOI:** 10.3389/fpsyg.2016.01181

**Published:** 2016-08-24

**Authors:** Colleen M. Ganley, Amanda L. McGraw

**Affiliations:** ^1^Department of Psychology, Florida State UniversityTallahassee, FL, USA; ^2^Florida Center for Research in Science, Technology, Engineering, and Math, Learning Systems Institute, Florida State UniversityTallahassee, FL, USA

**Keywords:** math anxiety, elementary-school children, math performance, gender, math attitudes

## Abstract

Although there is an extensive amount of research that examines the relation between math anxiety and math performance in adolescents and adults, little work has focused on this relation in young children. Recently more attention has been paid to the early development of math anxiety, and new measures have been created for use with this age group. In the present study, we report on the development and validation of a revised version of the Math Anxiety Scale for Young Children (MASYC; Harari et al., 2013). We conducted cognitive interviews with the 12 MASYC items with nine children and then administered the MASYC and five newly-developed items to 296 first-, second- and third-grade children. Results from cognitive interviews show that three of the items from the original scale were being systematically misinterpreted by young children. We present a revised measure (the MASYC-R) consisting of 13 items (eight original, five newly-developed) that shows strong evidence for reliability and validity. Results also showed that a small, but meaningful, proportion of children at this age show signs of high math anxiety. Validity of the MASYC-R was supported through correlations with a number of other factors, including general anxiety, math performance, and math attitudes. In addition, results suggest that a substantial proportion of the variance in math anxiety can be explained from these other variables together. The findings suggest that the MASYC-R is appropriate for use with young children and can help researchers to answer important questions about the nature and development of math anxiety at this age.

## Introduction

Math anxiety is defined as “a feeling of tension, apprehension, or fear that interferes with math performance” (Ashcraft, 2002, p. 181) and is a critical predictor of a number of math-related outcomes including math performance and the avoidance of math coursework (e.g., Richardson and Suinn, 1972; Wigfield and Meece, 1988; Ashcraft, 2002; Beilock, 2008). Despite the fact that we know math anxiety has important consequences, most of the current research on math anxiety has focused on adolescents and adults, with less research focusing on children, especially children at young ages. Fortunately, over the last decade there has been more interest in understanding how and when math anxiety develops, and researchers are beginning to examine math anxiety earlier in development (Wu et al., 2012; Harari et al., 2013; Jameson, 2013; Ramirez et al., 2013; Dowker et al., 2016). Importantly, this recent work suggests that math anxiety may begin to develop in children quite early (Jameson, 2013; Ramirez et al., 2013), which has important implications for later development, as research has found that math anxiety is fairly stable over time (Ma and Xu, 2004; Krinzinger et al., 2009; Cargnelutti et al., 2016). Critically, it is not just enough to measure math anxiety in young children, but also to test some of the theories of math anxiety that have been tested with adults. For example, it is critical to gain a better understanding of whether relations we consistently find with adolescents and adults (e.g., relations between math anxiety and other math attitudes and performance, relations between math anxiety and working memory) hold with younger students.

To better understand the development of math anxiety, we must have measures that allow us to make valid inferences about the math anxiety of young children. With this recent focus on math anxiety at younger ages, a number of new math anxiety measures have been developed for children of this age (first through third grade, or ages 7–10); however, there has been limited work examining the reliability and validity of these newly-developed measures. In the present study we examine the reliability and validity of one of these measures, the Math Anxiety Scale for Young Children (MASYC; Harari et al., 2013), as well as a revised version of the MASYC that we developed (MASYC-R). To assess the reliability of the scale, we examine the internal consistency and item-total correlations, and conduct confirmatory factor analyses (CFAs). To examine the validity of the scale we conduct cognitive interviews and examine how the scale is related to other critical variables including other math attitudes (math confidence, math interest, math importance), general anxiety, gender, and math performance. We also examine how these other variables together predict math anxiety to identify the most critical variables for better understanding math anxiety.

### The structure of math anxiety

Researchers have often conceptualized and measured math anxiety as a multidimensional construct. One conceptualization is that it includes emotionality, which is the physiological aspect of anxiety (e.g., palms sweating, heart racing) and worry, which is the cognitive aspect of anxiety (e.g., worried thoughts, racing thoughts; Liebert and Morris, 1967; Wigfield and Meece, 1988). In other research, it is conceptualized as involving two different components: math learning anxiety, which is anxiety often felt in the classroom or while doing math, and math evaluation anxiety, or anxiety felt during tests or while doing math in front of others (e.g., Plake and Parker, 1982; Hopko et al., 2003).

Recently researchers have used factor analyses to examine whether or not math anxiety in young children is a multidimensional construct. A number of researchers have found that multiple factors can be identified within this age group (e.g., Gierl and Bisanz, 1995; Wu et al., 2012; Harari et al., 2013; Jameson, 2013). For example, fitting with the Liebert and Morris (1967) emotionality/worry distinction, Harari et al. (2013) found that their scale, the MASYC, was best modeled by three factors: negative reactions (similar to emotionality), worry, and numerical confidence (reverse coded). Three other studies found subscales that map onto the math learning anxiety and math evaluation anxiety subscales found with adolescents and adults. With second and third graders, Wu et al. (2012) identified these two factors (which they called “numerical processing anxiety” and “situational and performance anxiety”). With a third-grade sample, Gierl and Bisanz (1995) found two similar factors, which they named “mathematics problem-solving anxiety” and “mathematics test anxiety.” In a sample of first- through fifth-grade children, Jameson (2013) found that their scale had three subscales: general math anxiety, math performance anxiety, and math error anxiety, the last of which both represent types of math evaluation anxiety. These results suggest that math anxiety may be a multidimensional construct, even at a young age, and that these dimensions may map onto those found with adolescents and adults.

### Challenges for measuring math anxiety in young children

Developing measures for young children comes with many challenges, regardless of the construct being measured (e.g., Besenski et al., 2007). There are also specific challenges to measuring emotions in general (e.g., Denham et al., 2009), and the construct of anxiety specifically in this age group (e.g., Schniering et al., 2000). These challenges are likely one of the reasons there has been less work on math anxiety with younger children. In the recent work in this area, the six scales that have been developed vary along a number of dimensions (as outlined in Table 1) and each of these authors had to make different decisions to address the challenges that come with measuring math anxiety with this age group.

**Table 1:** **Summary of currently available math anxiety measures for young children**.

**Measure title**	**# items**	**Rating scale**	**Sample item**	**α**	**Factors**	**Sample (grade and age range)**
Mathematics Anxiety Scale for Young Children (MASYC) a. Harari et al. (2013) b. Vukovic et al. (2013)	12	Yes Kind of Not really No	Math gives me a stomachache. (1 item is a math problem)	a. 0.70 b. 0.80	a. 3: negative reactions (α = 0.70), numerical confidence (α = 0.72), worry (α = 0.67) b. Treated as unidimensional	a. 106 1st graders (~7 years) b. 113 2nd graders (~8 years)
Children's Anxiety in Math Scale (CAMS) a. Jameson (2013) b. Jameson (2014)	16	5-point pictorial or verbal	When I solve math problems, I feel: 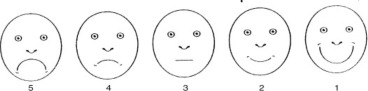 Faces from Buchanan and Niven (2002) (0 items are math problems)	a. 0.86 b. NR	a. 3: general math anxiety (α = 0.81), math performance anxiety (α = 0.73), math error anxiety (α = 0.74) b. Treated as unidimensional	a. 438 1st through 5th graders (~7 years to ~11 years) b. 91 2nd graders (~8 years)
Child Math Anxiety Questionnaire (CMAQ) a. Ramirez et al. (2013)	8	16-point sliding pictorial	How do you feel when taking a big test in your math class? Sliding scale with 3 faces from nervous to calm (4 items are math problems)	a. 0.55	a. Treated as unidimensional	a. 146 1st and 2nd graders (~7 years)
CMAQ-R a. Ramirez et al. (2016)	16	5-point pictorial	Being called on to explain a math problem on the board. 5 faces on scale; Faces not shown (some items are math problems)	a. 0.83	a. Treated as unidimensional	a. 564 1st and 2nd graders (~7 years)
Math Anxiety Questionnaire (MAQ)—Scales C and D a. Thomas and Dowker (2000) b. Krinzinger et al. (2007) c. Krinzinger et al. (2009)	14	5-point pictorial	How happy or unhappy are you if you have problems with written calculations 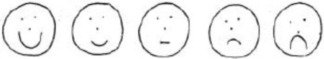 *very happy* to *very unhappy* How worried are you if you have problems with written calculations 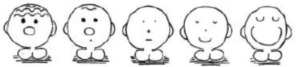 *very worried* to *very relaxed* (0 items are math problems)	a. NR b. 0.83−0.91 c. NR	a. NR b. Used as subscales and as 2 scales combined c. Used as subscales and as 2 scales combined	a. 6–9 years olds b. 450 1st through 3rd graders (6-9 years) c. 140 1st graders (~7.5 years)
Scale for Early Math Anxiety (SEMA) a. Wu et al. (2012) b. Wu et al. (2014) c. Cargnelutti et al. (2016)	20	5-point pictorial or verbal	You are in math class and your teacher is about to teach something new. 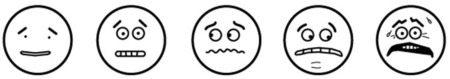 Verbal: *not at all nervous* to *very very nervous* (10 items are math problems)	a. 0.87 b. NR c. 0.86, 0.87	a. 2: numerical processing anxiety (α = 0.80), situational and performance anxiety (α = 0.77) b. Treated as unidimensional c. Treated as unidimensional	a. 162 2nd and 3rd graders (~7–8 years) b. 366 2nd and 3rd graders (~7–8 years) c. 80 2nd graders (~7 years) followed to 3rd grade

*NR, not reported*.

First, an issue that is relevant for all measures for this age group is that vocabulary and reading demands must be considered. If a survey is to be given to children in early elementary school, the items must not contain vocabulary that students will not understand. This is important to consider both for the words that attempt to capture anxiety (e.g., using nervous instead of anxious) and for additional words used in the sentence. It is also important to have items that have a simple sentence structure so that the items are at an appropriate reading level for students.

Second, to increase the research utility of a scale, it should be both short and able to be group administered. Generally, when we measure math anxiety we also measure a number of other constructs as well, thus a short measure is more useful so that we do not over-burden young children when collecting data. Currently, available measures range from 8 to 20 items (see Table 1) and it is important to balance the need to have a small number of items and the need to have good reliability. In regard to group administration, it is much more efficient for researchers if a scale can be administered to an entire class of students at once instead of requiring individual administration because this decreases the use of classroom instructional time for data collection.

The remaining issues are relevant due to issues that arise when measuring complex constructs in young children, as well as in measuring anxiety in general. First, it is important to ensure that the concept of anxiety is presented in a way that makes sense to young children. Some of the idiomatic ways in which adults talk about anxiety, such as sweaty palms, feeling sick to your stomach, etc., may not be well understood by young children. If they have not heard a particular phrase that is included in an item, they may not be able to figure out what the item is asking them, and may answer in a way that is inconsistent with the intended meaning of the item.

Last, the rating scale must make sense to the students and match the construct of anxiety. Traditional Likert scales go from *strongly disagree* to *strongly agree*, and the widely-used Math Anxiety Rating Scale (MARS) goes from *not anxious* to *very anxious* (MARS; Richardson and Suinn, 1972), choices which are not likely to make sense to young children. The MARS-E (a version of the MARS for upper elementary school children) goes from *not at all nervous* to *very, very nervous*, which works with older children (Suinn et al., 1988). With young children, a number of alternative strategies have been used (see Table 1), with most researchers relying on pictorial scales (e.g., Thomas and Dowker, 2000; Wu et al., 2012; Jameson, 2013; Ramirez et al., 2013, 2016), or simpler word choices (i.e., *yes, kind of*, *not really, no*; Harari et al., 2013).

The pictorial scales present some issues, including whether children can accurately interpret the pictures, as suggested by Krinzinger et al. (2009). In addition, choosing pictures that represent a scale from anxiety to no anxiety has proved challenging. Researchers have used faces that range from a frown to a smile (Thomas and Dowker, 2000; Jameson, 2013; see Table 1), faces that range from a nervous face to a smile (Thomas and Dowker, 2000), or faces that range from nervous to calm (Ramirez et al., 2013). These differences lend themselves to a theoretical debate about what exactly math anxiety *is* and what it means to *not* have math anxiety. We question whether math anxiety can be accurately represented as a frown, and a lack of math anxiety, a smile, as these seem to be more of a representation of student interest, liking, or enjoyment than math anxiety. A combination of pictorial and simple answer choices have been used by some researchers in an attempt to alleviate the concerns with the interpretation of pictorial scales (Wu et al., 2012; Jameson, 2013), however this may be more cognitively demanding for students as they must keep track of both representations.

For the current study, we selected the MASYC (Harari et al., 2013) as our measure and made revisions to it for potential improvement. We chose to start from this measure for four reasons. First, the rating scale was not pictorial, thus avoiding potential pitfalls of trying to determine how to represent anxiety (and lack of anxiety) as faces. Their rating scale is, instead, *yes, kind of*, *not really*, and *no*, which are very simple words for children to understand. Second, this scale was one of the shorter scales (12 items), while still maintaining acceptable reliability (α = 0.70, Harari et al., 2013; α = 0.80, Vukovic et al., 2013). Third, we were planning to use the scale across multiple grade levels and this scale did not have many items about specific math problems, unlike some of the other scales. This is important to consider because it could be that older students appear to be less math anxious simply because the math questions they are asked to reflect on are easier problems for them, and therefore they do not induce math anxiety. This would make the items difficult to use both cross-sectionally and longitudinally. Fourth, in some of our past research (Ganley and Vasilyeva, 2014), we were interested in specifically the worry component of anxiety, however, in some other research, the emotionality component of anxiety has been found to be more related to math performance (Wigfield and Meece, 1988; Ho et al., 2000; Harari et al., 2013), and therefore we wanted to use a scale that measured both aspects.

At the same time, we believed that we could improve on this measure for a few reasons. First, we were unsure if the wording of some of the items was appropriate for young children. Second, one of the items did ask about a specific math problem, so we wanted to test whether there were differences in responses across grades. Third, a number of the items asked students about if they “liked” different math activities, as opposed to if they felt nervous or calm, and these all loaded on one factor (numerical confidence), therefore it is unclear whether this is truly asking about math anxiety or about interest or something else entirely. Fourth, because we were interested in emotionality and worry based on our own and others' past work (Wigfield and Meece, 1988; Ho et al., 2000; Ganley and Vasilyeva, 2014), we wanted to include more items that map onto these factors. Thus, we believed this scale was a good starting point for developing a revised version that addressed some of these potential issues.

### The correlates of math anxiety

There is a large body of literature examining factors related to math anxiety. In our review of the literature we focus on research with children or adolescents with a greater emphasis on the research conducted with young children (first through third grades), as that is the population examined in the current study. We address each of these relations with math anxiety in turn, and also address potential gender differences in math anxiety.

#### Math performance

It is well documented that anxiety can be detrimental to performance. In particular, there is a large body of research that investigates math anxiety and its relation with math achievement, with estimates of the effect size for this relation being small to moderate in meta-analyses (*r* = −0.34 for grades 5–12, Hembree, 1990; *r* = −0.27 for grades 4–12, Ma, 1999). A number of researchers suggest that math anxiety is detrimental to math performance because anxiety consumes working memory resources needed to complete the math task, which in turn diminishes task performance (Eysenck and Calvo, 1992; Ashcraft and Kirk, 2001; Eysenck et al., 2007; Beilock, 2008; Ganley and Vasilyeva, 2014). This has been documented with a variety of math tasks ranging from challenging multi-step modular arithmetic to simple tasks like counting objects (Maloney et al., 2010; Mattarella-Micke et al., 2011). Importantly, research suggests that there is likely a bidirectional relation between math anxiety and performance, such that anxiety has situational detrimental impacts on performance, and that poor performance has long-term negative impacts on math anxiety, potentially through math avoidance (Ma, 1999; Ashcraft, 2002; Ma and Xu, 2004; Ganley and Vasilyeva, 2014; Carey et al., 2015).

In research with young children, the findings in regard to the relation between math anxiety and math performance are less consistent than they are with older children. Some research finds a relation, though usually small in size with correlations between −0.19 and −0.61 across studies, with most correlations in the −0.2 to −0.3 range (Gierl and Bisanz, 1995; Wu et al., 2012, 2014; Harari et al., 2013; Jameson, 2013; Ramirez et al., 2013, 2016; Vukovic et al., 2013; Cargnelutti et al., 2016), and others find no relation for some or all measures of math performance (Thomas and Dowker, 2000; Krinzinger et al., 2007, 2009; Harari et al., 2013; Vukovic et al., 2013; Cargnelutti et al., 2016). With the MASYC, Harari et al. (2013) found that negative reactions and numerical confidence were related to some types of math performance, but worry was unrelated. Thus, we can test if we find a similar pattern across factors.

#### General anxiety

Math anxiety can be conceptualized as anxiety that is specific to mathematics (Dowker et al., 2016), and therefore it is not surprising that it is often found to be moderately to strongly correlated with general anxiety (e.g., Hembree, 1990; Zettle and Raines, 2000; Wu et al., 2014). In the work with young children, the evidence is mixed, however, as three studies found that math anxiety was correlated with general anxiety (Wu et al., 2014; Cargnelutti et al., 2016; Hill et al., 2016) and two studies found no relation (Gierl and Bisanz, 1995; Wu et al., 2012). Hence, it is unclear from the literature how math anxiety and general anxiety may be related for young children. If they are related, then it is important to both (1) consider general anxiety when examining how math anxiety is related with other constructs to isolate the unique aspects of math anxiety over and above general anxiety (e.g., Hembree, 1990; Wu et al., 2012, 2014), and (2) examine how much unique variance in math anxiety can be attributed to general anxiety.

#### Math confidence

Researchers often find a moderate to large negative correlation between math anxiety and math confidence (Hembree, 1990; Meece et al., 1990; Goetz et al., 2010; Ganley and Vasilyeva, 2011; Ahmed et al., 2012), even in young children (Gierl and Bisanz, 1995; Krinzinger et al., 2009; Harari et al., 2013; Jameson, 2014). It could be argued that these are two poles to the same construct. In fact, Harari et al. (2013) include numerical confidence in their scale as the opposite of math anxiety. However, conceptually, it also seems that anxiety involves certain components that are not necessarily part of the construct of confidence. Specifically, as noted earlier, anxiety involves both physiological and cognitive reactions (i.e., emotionality and worry; Liebert and Morris, 1967) that would not necessarily be defined only as a lack of confidence. In line with this, Harari et al. (2013) found that their numerical confidence subscale was not correlated with the other aspects of math anxiety (*r*s = 0.02 and 0.16 with negative reactions and worry, respectively). Based on past research, it likely that relations between math anxiety and confidence exist at this age, but it is unclear how strong they are.

#### Math interest and importance

Some research has examined whether math anxiety is related to other math attitudes like math interest and math importance. Research has found a moderate negative relation between math anxiety and math interest (Wigfield and Meece, 1988; Hembree, 1990; Luo et al., 2009; Goetz et al., 2010; Harari et al., 2013) and math importance (Wigfield and Meece, 1988; Meece et al., 1990; Graham and Morales-Chicas, 2015; for an exception see Gierl and Bisanz, 1995). Harari et al. (2013) and Krinzinger et al. (2009) are the only studies to examine the relation between math interest and anxiety in students prior to middle school, and they both combined math interest with confidence (and found a relation). However, it is unclear if these results are only due to the inclusion of confidence items, thus more research addressing interest separately in young children is needed.

#### Gender

There is a large body of literature examining whether there are gender differences in math anxiety, but most of this work is with adolescents and adults. This work consistently finds small but statistically significant gender differences in math anxiety, with girls having higher levels of math anxiety than boys (e.g., Hyde et al., 1990; Miller and Bichsel, 2004; Frenzel et al., 2007; Ganley and Vasilyeva, 2014). However, research with elementary school children has more inconsistent results, with one study finding gender differences in math anxiety (Hill et al., 2016) and others finding no differences (Gierl and Bisanz, 1995; Harari et al., 2013; Ramirez et al., 2013; Jameson, 2014). Thus, it is unclear whether gender differences in math anxiety are apparent this early in development.

### The present research

In the present study we examined the reliability and validity of the MASYC developed by Harari et al. (2013), developed new scale items, and created a revised version of the MASYC. Prior to selecting new items and administering the scales to children, we conducted cognitive interviews to examine the validity of the items on the MASYC and to help develop the new items. We examined the reliability and structure of the original and newer scale by looking at internal consistencies (Cronbach's alphas) and item-total correlations and conducting a series of CFAs using the three factors empirically derived by Harari et al. (2013) as a guide. We then used information from cognitive interviews, and item-total correlations and factor loadings, to make decisions about the inclusion or exclusion of scale items. Our goal was to create a revised scale with stronger validity evidence and higher reliability than the original scale and that also included more items. Additional items were needed on the negative reactions (i.e., emotionality) and worry subscales, which contained three and four items, respectively, in the original version of the scale. In addition, relations with math confidence, math interest, math importance, general anxiety, math performance, and gender were examined to assess whether math anxiety was related to these variables in expected ways, to support the convergent validity of the scale. These are also conceptualized as research questions on their own, as these relations have not been explored extensively in early elementary-school children. We also conducted a regression analysis predicting math anxiety from these correlates to assess which variables are the best predictors of math anxiety and to examine how much of the variance in math anxiety can be explained by these variables altogether.

This study builds off past work and fills a number of gaps in the literature. First, to ensure that we are using a valid measure of math anxiety, we developed new items and conducted cognitive interviews to assess student understanding of the items. We then chose the new items based on the feedback we received from students and used data from cognitive interviews to make decisions about item inclusion during the factor analytic process with data from our main sample of almost 300 students. Second, we measured general anxiety, which allowed us to examine the relation between math anxiety and general anxiety and to examine the correlation between math anxiety and other constructs after we accounted for general anxiety. Third, much of the research in this area has been conducted with relatively small samples of students (~150 or less). Therefore, more work is needed with large and diverse samples of students.

## Methods

### Cognitive interview participants, measures, and procedure

Prior to collecting data with the entire sample, we conducted cognitive interviews with a sample of nine children (six in first grade, three in second grade). These students were recruited from a university family registry, which included children from a number of area schools. Students came into our research laboratory and worked one-on-one with a researcher for ~45 min completing all of the measures listed below in one session. Interviewers had at least a bachelor's degree, attended 2 h of interview training, and observed the first author conducting an interview before conducting interviews themselves. During the cognitive interviews we used the items from the MASYC and tried out nine newly-developed items from which we chose the final five new items that we gave to students in the main sample. During the cognitive interviews we read each item aloud (as was done in the original MASYC), asked students to respond, and then asked students to explain why they selected that choice. We then asked follow up questions to better understand why they made that choice (e.g., “What did you think of when you heard nervous?” “Do you know what butterflies in your stomach means?”). These results were used to help us make decisions first about which of the newly-developed items we should use, and second about which items from the original scale should be dropped. Because we wanted to test the structure of the original scale, we did not exclude or change any of the original items for our administration with the main sample based on cognitive interviews. Therefore, we only used the cognitive interview results to make decisions in the subsequent factor analytic process.

### Participants

Participants in the main sample were 296 students in the first through third grades (first grade *n* = 114, second grade *n* = 98, third grade *n* = 84) from two elementary schools in a city in the Southeast. The sample was ~55% percent male. The schools were racially and ethnically diverse (~45% White, ~35% Black, ~10% Hispanic, ~5% Asian, ~5% multi-race, on average) and socioeconomically diverse (~34% students qualify for free or reduced price lunch). Students were tested near the end of the school year. The average age of students at the time of testing was 7 years 3 months (*SD* = 5.4 months) for first-grade students, 8 years 3 months (*SD* = 4.6 months) for second-grade students, and 9 years 4 months (*SD* = 5.3 months) for third-grade students.

### Materials

For the majority of the math anxiety, math confidence, math interest, and math importance items, students responded on a four-point Likert Scale with choices of *yes, kind of, not really*, and *no*. However, there were five items (two from confidence, one from interest, and two from importance) that were on slightly different scales. For example for the math confidence item, “Compared to most of your other school subjects, how good are you at math?” the choices were *a lot better, a little better, a little worse*, and *a lot worse*. For the general anxiety scale, students responded to items on a four-point Likert Scale with choices of *always, often, sometimes*, and *never*. We reverse coded item responses when necessary so that higher numbers on the scale indicated higher agreement on that construct.

#### Math anxiety

The original MASYC and five newly-developed items were administered to students. The MASYC contains 12 items (see Table 2), five of which are worded in the opposite direction, such that agreement indicated a lack of anxiety (e.g., *I like being called on in math class*). The five new math anxiety items were developed by the researchers based on other math anxiety scales designed for use with children (Suinn et al., 1988; Chiu and Henry, 1990; Wu et al., 2012; Jameson, 2013; Ramirez et al., 2013) and based on the results of the cognitive interviews (see Table 2).

**Table 2:** **Item means, standard deviations, item-total correlations, and planned factors based on Harari et al. (2013) and theory**.

**#**	**Item**	**Mean**	**SD**	**Item-total r**	**Planned factor**		
					**Negative reactions**	**Numerical confidence**	**Worry**
**ORIGINAL MASYC ITEMS**							
1	Math gives me a stomachache.	1.32	0.85	0.54	X		
2	When it is time for math my head hurts.	1.49	0.97	0.61	X		
3	When it is time for math my heart beats fast.	1.93	1.21	0.47	X		
4	Figuring out if I have enough money to buy cookies and a drink is fun.^*^	1.81	1.09	0.09		X	
5	I like doing math problems on the board in front of the class.^*^	1.70	1.06	0.43		X	
6	I like to raise my hand in math class.^*^	1.71	1.02	0.52	(X)	X	
7	I like doing a math problem like this: 124 + 329^*^	1.76	1.12	0.29		X	
8	I like being called on in math class.^*^	1.67	1.04	0.52		X	
9	I get nervous about making a mistake in math.	2.54	1.27	0.44			X
10	When the teacher calls on me to tell my answer to the class, I get nervous.	2.06	1.22	0.62			X
11	I am scared in math class.	1.36	0.84	0.65	(X)		X
12	Getting out my math books makes me nervous.	1.29	0.78	0.56			X
**NEWLY-DEVELOPED ITEMS**							
14	I get worried before I take a math test.	2.38	1.26	0.52			X
16	My heart starts to beat fast if I have to do math in my head.	1.70	1.06	0.51	X		
17	I get nervous when my teacher is about to teach something new in math.	1.63	1.04	0.55			X
18	I get worried when I don't understand something in math.	2.40	1.22	0.50			X
21	I feel nervous when I am doing math.	1.57	1.01	0.69			X

Items are presented in the table in the same order as in Harari et al. (2013); Higher numbers indicate higher anxiety on a scale from 1 to 4;

* *Indicates than an item was reverse-coded such that higher numbers are equivalent to lower confidence. If an X is in parentheses it indicates that Harari et al found that it also loaded on this factor, but not as strongly as another factor*.

#### General anxiety

To measure general anxiety we used four items from the Spence Children's Anxiety Scale (Spence, 1997). One item was from the general domain (i.e., *I worry about things*) and three items were from the social domain (i.e., *I feel scared when I have to take a test, I worry that I will do badly at my school work*, and *I feel afraid if I have to talk in front of my class*). We selected these items because they generally have parallels with the items we ask (e.g., we ask about anxiety felt when talking in front of their math class and these ask about talking in front of class in general). The internal consistency (α) of this scale was 0.70 in the present sample.

#### Math confidence

Students were asked five questions about their math confidence (e.g., *I am good at math*). These items were adapted from Fredricks and Eccles (2002). The internal consistency (α) of this scale was 0.68 in the present sample.

#### Math interest

Students were asked three questions about their mathematical interest (e.g., *I like doing math*), which were adapted from Fredricks and Eccles (2002). One item was removed because it did not correlate well with the other two items. The internal consistency (α) of the remaining two items was 0.75 in the present sample.

#### Math importance

Students were asked four questions about their perception of the importance of math (e.g., *Being good at math is important to me*), which were adapted from Fredricks and Eccles (2002). This scale's internal consistency (α) was 0.73 in the present sample.

#### Math performance

Students completed a researcher-developed 17-item math test that covered topics in measurement, number, and algebra that were appropriate for first- through third-grade students. Internal consistency (α) of the test was 0.83 in the present sample.

### Procedure

This study was carried out in accordance with the recommendations of the Declaration of Helsinki and approved by the Institutional Review Board at Florida State University. Written assent was obtained from all participants after written informed consent was obtained from a parent or guardian. For the main sample, testing took place over 2 days during the last month of the school year. The first day of testing consisted of assent procedures and the math assessment (35–40 min) and the second day of testing consisted of the math anxiety, general anxiety, confidence, interest, and importance scales (15–20 min). Instructions for each task were read to the students before they filled out the measures and all items were read aloud. To ensure that students understood what the measures were asking, they were given a practice problem before the math assessment and sample questions on an unrelated topic (e.g., I like pizza) before the math attitudes scales to demonstrate understanding of the Likert-style scale rating. Testers had at least a bachelor's degree, attended 2 h of training, and observed the first author testing a classroom before administering the assessments themselves.

Because the math test was administered on a different day than the other measures, we were missing data for 15 students who were absent on the math testing day, thus a sample of 281 students was used for analyses that involved math performance. We were missing gender data for 7 students, who were excluded for analyses that involved gender.

## Results

### Cognitive interview results

As a reminder, we conducted cognitive interviews with nine first- and second-grade students to assess if the children were interpreting items as intended. Results suggested that young children were misinterpreting a subset of the items. The results of the interviews are summarized in Table S1 in the Supplementary Material with examples of children's appropriate and inappropriate interpretations. Through these interviews, we identified three items from the MASYC and some of the newly-developed items as potentially problematic.

Based on the cognitive interviews, we decided to remove the three identified MASYC items from the scales (items 3, 4, and 12), which were each misinterpreted by over half of the students. For item 3, *When it is time for math my heart beats fast*, five of the nine children did not interpret the item in the intended way by either saying “Yes” because they had recess beforehand, or saying “Yes” because they get excited when it is time for math, which leads to their heart beating fast (see Table S1 for sample student responses). For item 4, *Figuring out if I have enough money to buy cookies and a drink is fun*, eight of the nine students responded with an unintended interpretation. Most students made judgments about how much they would want to purchase cookies or a drink, without consideration of the mathematics involved in the statement. For item 12, *Getting out my math books makes me nervous*, five of the nine children did not interpret the item as intended for a number of different reasons including saying “No” because they do not have a math book or because their teacher hands out the math books.

For our newly-developed items, we dropped a few items during the cognitive interviews. We dropped item 13, *When it's time for math I get butterflies in my stomach*, due to three of the six students not knowing what this phrase meant. We dropped item 19, *I get nervous when I see a page of math problems that I need to solve*, because all students said no and it was not very different from other items. We dropped an additional two items that were similar to other items because we did not want to overburden children with too many items (dropped item 15 because it was similar to 17, dropped item 20 because it was similar to 14). We also added item 21, *I feel nervous when I am doing math*, partway through cognitive interviews because it was very direct and we had noticed that students were better at accurately interpreting items that were more direct. Children tended to have more difficulty with less direct items because they got caught up in the details of the items (e.g., focusing on “cookies” instead of “figuring out”). This left us with the final five newly-developed items (items 14, 16, 17, 18, and 21 in Table 2 and Table S1 in the Supplementary Material).

### Scale reliability and factor structure

Descriptive statistics for all variables by grade level are presented in Table 3. Prior to examining the scale structure of the math anxiety scale, we examined basic reliability information. We examined the internal consistency using Cronbach's alpha for the entire 12-item (MASYC) and 17-item (MASYC + 5 new items) scales and item-total correlations. The internal consistencies (α) were 0.80 for the 12-item scale and 0.87 for the 17-item scale. Item-total correlations are presented in Table 2. Inspection of these correlations indicate that item 4 (*Figuring out if I have enough money to buy cookies and a drink is fun*) had a much lower item-total correlation than the other items, with an item-total correlation of 0.13 for the 12-item scale and 0.09 for the 17-item scale. Even when considered with only the 5 items in its factor (numerical confidence), the item-total correlation was 0.19. Analyses by grade show that this item was especially problematic at first grade, with a negative item-total correlation (−0.06 12-item, −0.12 17-item), but still did not perform especially well at the second and third grade with item-total correlations of 0.25 and 0.21 in second grade and 0.35 and 0.32 in third grade, for the 12- and 17-item scales, respectively. This item was also the worst performing item in the cognitive interviews, with eight of the nine students misinterpreting it, and thus was excluded as part of the factor analysis process.

**Table 3:** **Descriptive statistics for key variables by grade level**.

	**First grade *n* = 114**		**Second grade *n* = 98**		**Third grade *n* = 84**	
	**Mean**	***SD***	**Mean**	***SD***	**Mean**	***SD***
MASYC negative reactions	1.69^a^	0.91	1.45^b^	0.64	1.59^c^	0.78
MASYC numerical confidence	1.83	0.72	1.61	0.64	1.74	0.69
MASYC worry	1.83	0.80	1.73	0.69	1.88	0.72
MASYC-R negative reactions	1.59	0.82	1.34	0.58	1.45	0.66
MASYC-R numerical confidence	1.77	0.89	1.55	0.76	1.75	0.88
MASYC-R worry	2.12	0.84	1.99	0.78	2.20	0.85
General anxiety	1.95	0.72	1.88	0.72	2.06	0.72
Math confidence	3.56^a^	0.44	3.57^a^	0.45	3.35^b^	0.58
Math interest	3.28	0.86	3.24	0.91	3.21	0.91
Math importance	3.45	0.56	3.55	0.58	3.51	0.56
Math performance	32.80^a^	19.63	47.92^b^	19.51	69.77^c^	17.82

*The statistics for the anxiety factors are based on the original scale metric (1–4) and are not factor scores. The numerical confidence scale is reverse-coded such that higher numbers are equivalent to lower confidence. The sample sizes for math performance are: first grade = 106, second grade = 96, third grade = 79. If a score has a superscript of a different letter from another, those were found to be statistically significantly different from one another. If there are no superscripts there was no difference*.

Item 7 (*I like doing a math problem like this: 124* + *329*) also did not have a high item-total correlation, with an item-total correlation of 0.29 for both the 12- and 17-item scales. When considered only with the 5 items in the numerical confidence factor, the item-total correlation was 0.28. We had concerns about this item initially because we thought it might not be appropriate for use cross-sectionally across grades or longitudinally because students in different grades would have different judgments based on what they have been exposed to in school. Importantly, our results showed that this item was the only item of the 17 items that differed across grades, *F*_(2, 292)_ = 5.23, *p* = 0.006, with first graders reporting higher anxiety ratings (*M* = 2.02, *SD* = 1.28) than second (*M* = 1.55, *SD* = 0.99) and third graders (*M* = 1.66, *SD* = 0.98), who did not differ from one another. Based on these grade differences and the low item-total correlations, we decided to remove this item during the factor analytic process. All remaining item total correlations were greater than 0.40 for the 12-item and 17-item scales.

We then proceeded to conduct a number of CFAs to determine whether the factor structure found by Harari et al. (2013) was a good fit to our data and to test alternative models based on cognitive interview data and reliability data from the present sample. All analyses were conducted in MPlus (Muthén and Muthén, 1998-2012). Responses on the math anxiety items were treated as categorical because there were only four response options. The model was estimated with weighted least squares estimation (WLSMV), which is considered the best estimator for categorical data, especially data with high skew or kurtosis (Brown, 2006). We evaluated model fit using guidelines provided by MacCallum et al. (1996) for assessing Root Mean Square Error of Approximation (RMSEA) estimates and guidelines provided by Hu and Bentler (1999) for assessing Comparative Fit Index (CFI) and Tucker Lewis Index (TLI). We interpreted RMSEA values < 0.05 and CFI and TLI values > 0.95 as indicators of close fit. Fit indices for all models are displayed in Table 4. We estimated intraclass correlations for each of the items across classrooms and they ranged from 0.01 to 0.07, suggesting that classrooms were only accounting for a small amount of variance, and that we could run more parsimonious models without clustering by classroom.

**Table 4:** **Goodness of fit statistics for models**.

**#**	**Model**	***df***	**χ^2^**	**RMSEA**	**90% CI**	**CFI**	**TLI**	**α**	**NR α**	**NC α**	**W α**
**ORIGINAL MASYC (12 ITEMS)**											
1	Univariate	54	212.69	0.100	0.086–0.114	0.898	0.876	0.80			
2	3-factor	51	92.32	0.052	0.035–0.069	0.973	0.966		0.69	0.66	0.67
**NEW FULL SCALE (17 ITEMS)**											
3	Univariate	119	355.86	0.082	0.072–0.092	0.914	0.901	0.87			
4	3-factor (item 11 on NR)	116	180.57	0.043	0.031–0.055	0.977	0.972		0.78	0.66	0.81
**NEW FULL SCALE WITH MISUNDERSTOOD AND MATH PROBLEM ITEMS REMOVED (MASYC-R; 13 ITEMS)**											
5	Univariate	65	255.76	0.100	0.087–0.113	0.916	0.899	0.87			
6^a^	3-factor (item 11 on NR)	62	98.16	0.044	0.027–0.060	0.984	0.980		0.76	0.75	0.80
7	2-factor (NR and W combined)	64	106.78	0.048	0.031–0.063	0.981	0.977		0.86	0.75	

df, degrees of freedom; χ^2^, chi-square statistic; RMSEA, root mean square error of approximation; 90% CI, 90% confidence interval around the RMSEA; CFI, comparative fit index; TLI, Tucker-Lewis index; α, Cronbach's alpha; NR, Negative Reactions; NC, Numerical Confidence; W, Worry

a *Model 6 was identified as the final model*.

**Figure 1:**
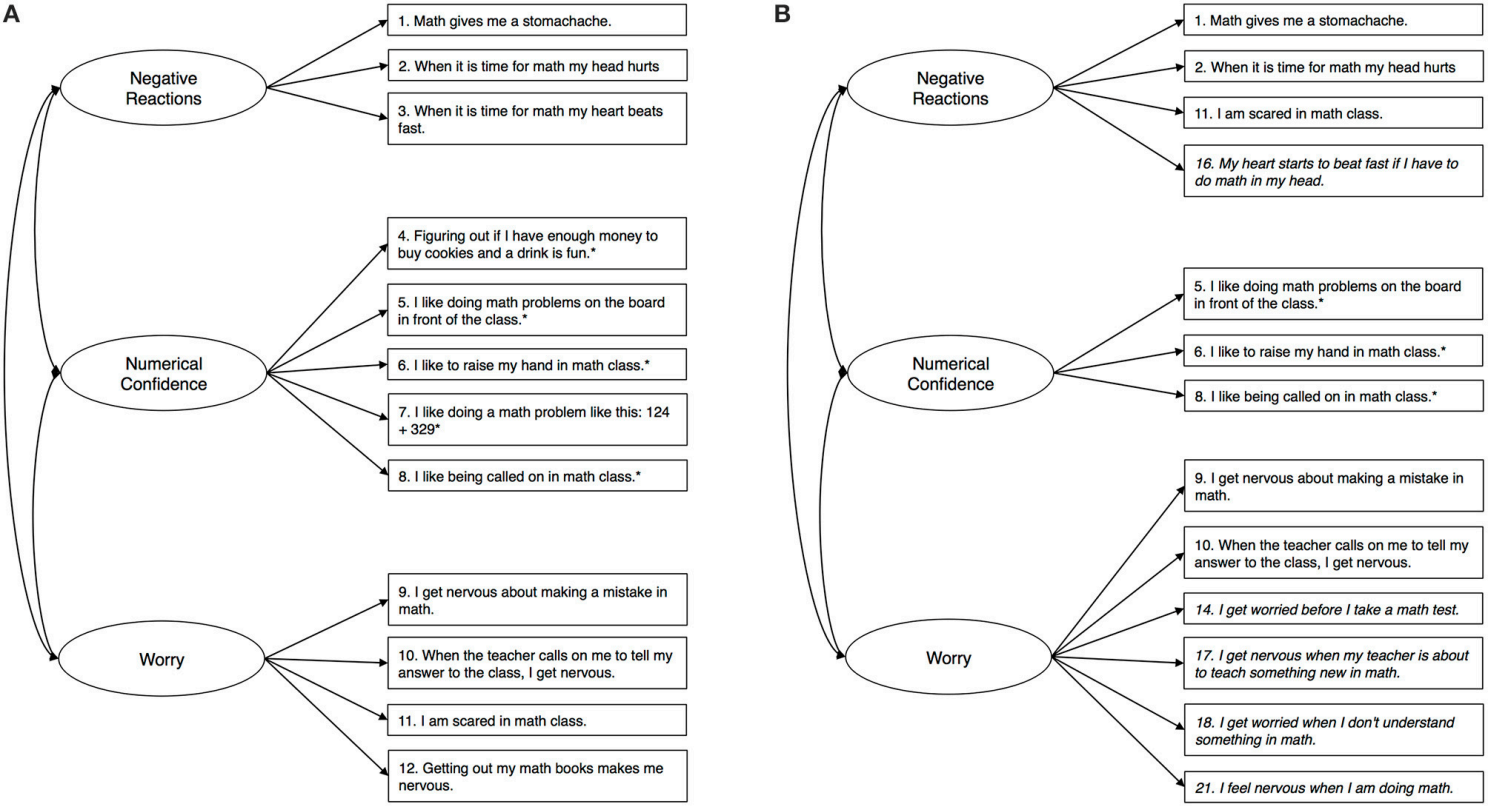
**(A)** Shows the original structure of the MASYC as proposed by Harari et al. (2013). **(B)** Shows the final factor structure of the MASYC-R, including the newly-developed items (in italics). ^*^Indicates a reverse coded item.

To examine the structure of the scale we began with the original MASYC items and modeled them as a univariate solution and according to their three-factor solution (see Figure 1). Fit statistics for the univariate model showed that the model did not meet any of the criteria for close fit [$\chi_{\left(54\right)}^{2}$ = 212.69, *p* < 0.001, RMSEA = 0.100 (90% CI = 0.086 − 0.114], CFI = 0.898, and TLI = 0.876). We then estimated the three-factor model proposed by Harari et al. (2013). As seen in Table 2 and Figure 1, the first factor was negative reactions [items 1, 2, 3, (6, 11)], the second was numerical confidence [items 4, 5, (6), 7, 8], and the third was worry, [items 9, 10, (11), 12]. Items in parentheses are items that Harari et al. found to load on two factors. We allowed these items to load on two factors in our model and then tested them only allowing the items to load on the factor that they had the highest loading on in Harari et al. Results were very similar with these two strategies, so we proceeded with the model that did not have cross-loadings for the sake of parsimony and usability. The three-factor model met close fit criteria for the CFI and TLI, but not for the RMSEA $\chi_{\left(51\right)}^{2}$ = 92.32, *p* < 0.001, RMSEA = 0.052 (90% CI = 0.035 − 0.069), CFI = 0.97, and TLI = 0.97 (Table 4). Factor loadings for this model are displayed in Table 5.

**Table 5:** **Factor loadings for the original Harari et al. (2013) MASYC model and the final MASYC-R model**.

**#**	**Item**	**Harari et al. (****2013****) model CFA results**			**Final MASYC-R model CFA results**		
		**Negative reactions**	**Numerical confidence**	**Worry**	**Negative reactions**	**Numerical confidence**	**Worry**
**ORIGINAL MASYC ITEMS**							
1	Math gives me a stomachache.	0.85			0.80		
2	When it is time for math my head hurts.	0.89			0.80		
3	When it is time for math my heart beats fast.	0.64			–		
4	Figuring out if I have enough money to buy cookies and a drink is fun.^*^		0.22			–	
5	I like doing math problems on the board in front of the class.^*^		0.70			0.67	
6	I like to raise my hand in math class.^*^		0.81			0.80	
7	I like doing a math problem like this: 124 + 329^*^		0.41			–	
8	I like being called on in math class.^*^		0.87			0.89	
9	I get nervous about making a mistake in math.			0.53			0.60
10	When the teacher calls on me to tell my answer to the class, I get nervous.			0.74			0.80
11	I am scared in math class.			0.91	0.90		
12	Getting out my math books makes me nervous.			0.81			–
**NEWLY-DEVELOPED ITEMS**							
14	I get worried before I take a math test.						0.71
16	My heart starts to beat fast if I have to do math in my head.				0.68		
17	I get nervous when my teacher is about to teach something new in math.						0.73
18	I get worried when I don't understand something in math.						0.64
21	I feel nervous when I am doing math.						0.89
	Latent correlation with negative reactions		0.60	0.92		0.62	0.90
	Latent correlation with numerical confidence			0.68			0.62

*All factor loadings were significant at p < 0.001 with the exception of item 4, which was significant at p < 0.01*.

* *Indicates than an item was reverse-coded*.

Next, we added in the five items that we developed and examined the factor structure of these 17 items together. We then eliminated the MASYC items identified as problematic in the cognitive interviews and inter-item correlations/theory. In Table 2 we identify the factor that each of the new items we created were meant to load on (i.e., item 16 on negative reactions, items 14, 17, 18, and 21 on worry). We also want to mention here that based on the Liebert and Morris (1967) definitions of the types of anxiety, it seemed that item 11 (*I am scared in math class*) was a better conceptual fit with the negative reactions scale (Liebert and Morris's emotionality) than the worry scale because it gets more at a physiological fear response than a cognitive worry response. Empirical information from Harari et al.'s original paper support this potential change, as they found that item loadings were very similar for this item for the two factors (0.33 on negative reactions and 0.44 on worry). Thus, we tried this item in both locations, ultimately determining that it fit better both empirically and theoretically on the negative reactions scale than the worry scale and we therefore proceeded with this model when testing different factor structures^1^.

We began with parallel models to the original Harari et al. (2013) models but with all 17 items. The univariate model did not meet criteria for close fit, but the three-factor solution did, $\chi_{\left(116\right)}^{2}=$ 180.57, *p* < 0.001, RMSEA = 0.043 (90% CI = 0.031–0.055), CFI = 0.977, and TLI = 0.972. We then removed the MASYC items identified in cognitive interviews and with inter-item correlations/theory as potentially problematic (items 3, 4, 7, and 12). The fit indices for the resulting three-factor model were similar to those with all 17 items and also met criteria for close fit, [$\chi_{\left(62\right)}^{2}$ = 98.16, *p* < 0.001, RMSEA = 0.044 (90% CI = 0.027–0.060), CFI = 0.984, and TLI = 0.980] thus showing we could increase the validity without disrupting the reliability and factor structure^2^ of the measure (Table 5).

Latent correlations between factors in this model are *r* = 0.90 for negative reactions and worry, and *r* = 0.62 for numerical confidence with both negative reactions and worry. Because the correlation between negative reactions and worry was so high, we also tested a model with these two factors combined. Fit statistics still indicated close fit [$\chi_{\left(64\right)}^{2}$ = 106.78, *p* < 0.001, RMSEA = 0.048 (90% CI = 0.031–0.063), CFI = 0.981, and TLI = 0.977]. However, a χ^2^ difference test (using DIFFTEST in MPlus) showed that this model was a worse fitting model than the three-factor model [χ^2^ difference (*df* = 2) = 7.82, *p* = 0.02]. Therefore, the three-factor model was retained.

### Levels of math anxiety

It is important to better our understanding of the development of math anxiety to see how many children at this age can be identified as having high math anxiety. Our results suggest that there is a subset of students at this age showing elevated levels of math anxiety (Table 6). If instead of looking at factor scores, we examine the raw scores for students on the 1–4 scale, with a midpoint of 2.5, we see that even as early as first grade 13.2% of students are above the midpoint on negative reactions (8.0% at second, 12.0% at third), 17.6% are above the midpoint (indicating low confidence) on numerical confidence (15.2% at second, 20.3% at third), and 33.1% percent of students are above the midpoint on worry (25.5% at second, 37.1% at third). Smaller portions of students have what might be considered very high levels of math anxiety that are closer to the maximum of the scale (between 3.5 and 4) and there are more students at the extreme in worry (11.4% in first grade, 4.0% in second grade, 12.0% in third grade; see Table 6).

**Table 6:** **Percent of participants at different mean ranges in math anxiety on the MASYC-R across grade levels**.

**Mean**	**First grade**			**Second grade**			**Third grade**		
	**NR (%)**	**NC (%)**	**Worry (%)**	**NR (%)**	**NC (%)**	**Worry (%)**	**NR (%)**	**NC (%)**	**Worry (%)**
1–1.49	56.1	50.0	25.4	69.4	65.3	27.4	65.5	43.5	21.5
1.5–1.99	18.5	7.9	19.3	21.4	7.1	21.4	13.1	11.9	24.9
2–2.49	12.3	24.5	22.8	1.0	12.2	24.3	9.6	14.3	16.7
2.5–2.99	3.5	4.4	12.1	4.0	6.1	11.2	6.0	7.1	14.3
3–3.49	3.5	5.3	9.6	2.0	6.1	10.3	4.8	4.8	10.8
3.5–4	6.2	7.9	11.4	2.0	3.0	4.0	1.2	8.4	12.0
Below midpoint	86.9	82.4	67.5	91.8	84.6	73.1	88.2	69.7	63.1
Above midpoint	13.2	17.6	33.1	8.0	15.2	25.5	12.0	20.3	37.1

*NR, negative reactions; NC, numerical confidence. All items were rated on a scale from 1 to 4. The numerical confidence scale is reverse-coded such that higher numbers are equivalent to lower confidence. Some columns may not add to 100% due to rounding*.

### Validity

**Figure 2:**
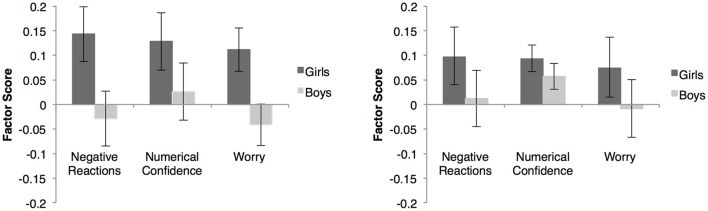
**This figure presents gender differences in factor scores for each math anxiety factor in the final MASYC-R model. (A)** shows unadjusted means and **(B)** shows adjusted means after accounting for general anxiety. Error bars represent standard errors.

To test the convergent validity of the subscales, we examined correlations among the factor scores for each of the original MASYC factors and each of the factors from the final MASYC-Revised (MASYC-R), with math confidence, math interest, math importance, and math performance, both with and without covarying out general anxiety (see Tables 7, 8). We also examined the size of the gender difference in math anxiety with and without covarying out general anxiety (Figure 2).

**Table 7:** **Correlations between math anxiety factors and other key variables**.

	**MASYC NR**	**MASYC NC**	**MASYC W**	**MASYC-R NR**	**MASYC-R NC**	**MASYC-R W**	**Gen anx**	**Math conf**	**Math int**	**Math imp**	**Math perf**
MASYC negative reactions	1										
MASYC numerical confidence	0.72^**^	1									
MASYC worry	0.98^**^	0.77^**^	1								
MASYC-R negative reactions	0.93^**^	0.71^**^	0.94^**^	1							
MASYC-R numerical confidence	0.70^**^	0.98^**^	0.76^**^	0.73^**^	1						
MASYC-R worry	0.87^**^	0.69^**^	0.91^**^	0.97^**^	0.73^**^	1					
General anxiety	0.54^**^	0.43^**^	0.57^**^	0.62^**^	0.45^**^	0.66^**^	1				
Math confidence	−0.47^**^	−0.50^**^	−0.51^**^	−0.53^**^	−0.51^**^	−0.54^**^	−0.51^**^	1			
Math interest	−0.52^**^	−0.55^**^	−0.53^**^	−0.52^**^	−0.54^**^	−0.52^**^	−0.39^**^	0.59^**^	1		
Math importance	−0.36^**^	−0.49^**^	−0.37^**^	−0.36^**^	−0.47^**^	−0.34^**^	−0.27^**^	0.44^**^	0.54^**^	1	
Math performance	−0.14^*^	−0.06	−0.13^*^	−0.19^**^	−0.08	−0.20^**^	−0.13^*^	0.11	0.08	−0.04	1

*Correlations with math performance are partial correlations in which grade is partialled out because there are large grade differences in math performance*.

* p < 0.05;

** *p < 0.01*.

**Table 8:** **Partial correlations between math anxiety and other key variables with general anxiety as a covariate**.

	**MASYC**			**MASYC-R**		
	**Negative reactions**	**Numerical confidence**	**Worry**	**Negative reactions**	**Numerical confidence**	**Worry**
Math confidence	−0.27^**^	−0.36^**^	−0.31^**^	−0.31^**^	−0.36^**^	−0.32^**^
Math interest	−0.40^**^	−0.46^**^	−0.41^**^	−0.39^**^	−0.45^**^	−0.38^**^
Math importance	−0.27^**^	−0.43^**^	−0.28^**^	−0.26^**^	−0.41^**^	−0.23^**^
Math performance	−0.09	−0.01	−0.07	−0.13^*^	−0.02	−0.15^*^

*Correlations with math performance are conducted while taking grade into account because there are large grade differences in math performance*.

* p < 0.05;

** *p < 0.01*.

All three latent factors for both the original MASYC and the MASYC-R were significantly positively correlated with general anxiety (0.43 < *r*s < 0.66), and significantly negatively correlated with math confidence (−0.47 < *r*s < −0.54), math interest (−0.52 < *r*s < −0.55), and math importance (−0.34 < *r*s < −0.49; see Table 7). When general anxiety was taken into account for the correlations with confidence, interest, and importance, the correlations were attenuated but still statistically significant (confidence: −0.27 < partial *r*s < −0.36; interest −0.38 < partial *r*s < −0.46; importance: −0.23 < partial *r*s < −0.43; see Table 8).

Results showed that for both the MASYC and the MASYC-R, the negative reactions (MASYC *r* = −0.14; MASYC-R *r* = −0.19) and worry factors (MASYC *r* = −0.13; MASYC-R *r* = −0.20) were correlated with math performance^3^. Numerical confidence was not correlated with math performance for either scale (MASYC *r* = −0.06; MASYC-R *r* = −0.07). When general anxiety was partialled out, the correlations between the math anxiety factors and math performance were attenuated, and the original MASYC negative reactions and worry factors were no longer significantly correlated with math performance (*r*s = −0.09 and −0.07, respectively), but the MASYC-R negative reactions and worry factors were still significantly correlated with math performance (*r*s = −0.13 and −0.15, respectively).

In regard to gender, results showed that girls (*n* = 135, *M* = 0.14, *SD* = 0.65) rated their math anxiety higher than did boys (*n* = 154, *M* = −0.03, *SD* = 0.69) on the negative reactions scale for the MASYC-R, *F*_(1, 287)_ = 4.69, *p* = 0.031, *d* = 0.26. Girls (*M* = 0.11, *SD* = 0.51) also rated their worry higher than did boys (*M* = −0.04, *SD* = 0.52) for the MASYC-R, *F*_(1, 287)_ = 6.30, *p* = 0.013, *d* = 0.30. However, when the small, but nonsignificant gender difference in general anxiety was taken into account, there was no longer a significant gender difference in either negative reactions, *F*_(1, 286)_ = 1.82, *p* = 0.18, *d* = 0.16, or worry, *F*_(1, 286)_ = 3.14, *p* = 0.08, *d* = 0.21. All other gender differences in the MASYC and MASYC-R math anxiety factors were not statistically significant with and without adjustment based on general anxiety.

### Predicting math anxiety

To understand what the best predictors of the construct of math anxiety are at early elementary school, we conducted a regression analysis in which we predicted math anxiety from general anxiety, math performance, and math attitudes. We conducted three hierarchical regression analyses, one for each MASYC-R factor, in which we first entered general anxiety and math performance, and then entered the three math attitude variables (math confidence, interest, and importance).

For negative reactions, the first model, with general anxiety and math performance, accounted for 39% of the variance. In this model, general anxiety (β = 0.61, *p* < 0.001), and math performance (β = −0.11, *p* = 0.02), were both significant predictors (see Table 9). When math attitudes were included, an additional 11% of the variance was explained, general anxiety and math performance remained significant, and math confidence (β = −0.14, *p* = 0.01) and math interest (β = −0.23, *p* < 0.001) were statistically significant predictors but math importance (β = −0.08, *p* = 0.14) was not.

**Table 9:** **Regression analysis result predicting MASYC-R factors: entire sample**.

	**Negative reactions**				**Numerical confidence**				**Worry**			
	**Model 1**		**Model 2**		**Model 1**		**Model 2**		**Model 1**		**Model 2**	
	**β**	**sr^2^**	**β**	**sr^2^**	**β**	**sr^2^**	**β**	**sr^2^**	**β**	**sr^2^**	**β**	**sr^2^**
General anxiety	0.61^**^	0.37	0.43^**^	0.14	0.44^**^	0.20	0.21^**^	0.03	0.64^**^	0.41	0.47^**^	0.16
Math performance	−0.11^*^	0.01	−0.12^**^	0.01	−0.02	< 0.01	−0.03	< 0.01	−0.10^*^	< 0.01	−0.10^*^	0.01
Math confidence			−0.14^*^	0.01			−0.16^*^	0.01			−0.16^**^	0.01
Math interest			−0.23^**^	0.03			−0.26^**^	0.04			−0.22^**^	0.03
Math importance			−0.08	< 0.01			−0.21^**^	0.03			−0.03	< 0.01
R^2^ change				0.11^**^				0.21^**^				0.10^**^
R^2^		0.39^**^		0.50^**^		0.20^**^		0.41^**^		0.43^**^		0.53^**^

* p < 0.05;

** *p < 0.01; n = 281*.

For numerical confidence, the first model, with general anxiety and math performance, accounted for 20% of the variance. In this model, general anxiety (β = 0.44, *p* < 0.001) was a statistically significant predictor, but math performance was not (β = −0.02, *p* = 0.69; see Table 9). When math attitudes were included, an additional 21% of the variance was explained, general anxiety remained significant, and math confidence (β = −0.16, *p* = 0.01), math interest (β = −0.26, *p* < 0.001) and math importance (β = −0.21, *p* < 0.001) were all statistically significant predictors.

For worry, the first model, with general anxiety and math performance, accounted for 43% of the variance. Both general anxiety (β = 0.64, *p* < 0.001) and math performance (β = −0.10, *p* = 0.04) were significant predictors (see Table 9). When math attitudes were included, an additional 10% of the variance was explained, general anxiety and math performance remained significant, and math confidence (β = −0.17, *p* = 0.004) and math interest (β = −0.22, *p* < 0.001) were statistically significant predictors, but math importance (β = −0.03, *p* = 0.50) was not.

We also conducted these regression analyses for each grade separately to test whether there were any differences in these patterns across development. The results of these analyses are reported in Tables 10–12. For negative reactions and worry, results show that general anxiety is a consistent strong predictor of math anxiety across grades. The pattern for math performance is less consistent, as math performance only relates to negative reactions in the third grade when math attitudes are also considered, and to worry in third grade both with and without attitudes considered. Math attitudes appear to be less important at the second grade, when none of the attitudes predict negative reactions or worry, and together do not explain a significant amount of variance. For first and third graders, different math attitudes appear to be important, with math interest being significant in first grade and math confidence and math importance being significant at third grade. We were better able to predict negative reactions and worry from general anxiety, math performance, and math attitudes for older students (*R*^2^ = 0.47 and 0.48 at first grade, 0.54, and 0.57 at second grade, and 0.67 and 0.69 at third grade, for negative reactions and worry, respectively).

**Table 10:** **Regression analysis results predicting MASYC-R factors: first grade**.

	**Negative reactions**				**Numerical confidence**				**Worry**			
	**Model 1**		**Model 2**		**Model 1**		**Model 2**		**Model 1**		**Model 2**	
	**β**	**sr^2^**	**β**	**sr^2^**	**β**	**sr^2^**	**β**	**sr^2^**	**β**	**sr^2^**	**β**	**sr^2^**
General anxiety	0.54^**^	0.29	0.39^**^	0.12	0.29^**^	0.08	0.08	< 0.01	0.57^**^	0.32	0.42^**^	0.14
Math performance	−0.08	< 0.01	−0.09	< 0.01	0.09	< 0.01	0.07	< 0.01	−0.08^*^	< 0.01	−0.09	< 0.01
Math confidence			−0.04	< 0.01			−0.20	0.03			−0.06	< 0.01
Math interest			−0.42^**^	0.12			−0.30^**^	0.06			−0.38^**^	0.10
Math importance			< 0.01	< 0.01			−0.13	0.01			−0.02	< 0.01
R^2^ change				0.17^**^				0.21^**^				0.14^**^
R^2^		0.31^**^		0.47^**^		0.09^**^		0.30^**^		0.33^**^		0.48^**^

* p < 0.05;

** *p < 0.01; n = 106*.

**Table 11:** **Regression analysis results predicting MASYC-R factors: second grade**.

	**Negative reactions**				**Numerical confidence**				**Worry**			
	**Model 1**		**Model 2**		**Model 1**		**Model 2**		**Model 1**		**Model 2**	
	**β**	**sr^2^**	**β**	**sr^2^**	**β**	**sr^2^**	**β**	**sr^2^**	**β**	**sr^2^**	**β**	**sr^2^**
General anxiety	0.70^**^	0.48	0.57^**^	0.23	0.59^**^	0.35	0.35^**^	0.09	0.73^**^	0.53	0.62^**^	0.28
Math performance	−0.08	< 0.01	−0.08	< 0.01	0.03	< 0.01	0.02	< 0.01	−0.08	< 0.01	−0.06	< 0.01
Math confidence			−0.16	0.01			−0.15	0.01			−0.15	0.01
Math interest			−0.06	< 0.01			−0.20	0.02			−0.09	< 0.01
Math importance			−0.06	< 0.01			−0.20^*^	0.02			−0.02	< 0.01
R^2^ change				0.04				0.16^**^				0.03
R^2^		0.49^**^		0.54^**^		0.35^**^		0.50^**^		0.54^**^		0.57^**^

* p < 0.05;

** *p < 0.01; n = 96*.

**Table 12:** **Regression analysis result predicting MASYC-R factors: third grade**.

	**Negative reactions**				**Numerical confidence**				**Worry**			
	**Model 1**		**Model 2**		**Model 1**		**Model 2**		**Model 1**		**Model 2**	
	**β**	**sr2**	**β**	**sr^2^**	**β**	**sr^2^**	**β**	**sr^2^**	**β**	**sr^2^**	**β**	**sr^2^**
General anxiety	0.58^**^	0.31	0.32^**^	0.07	0.48^**^	0.21	0.28^**^	0.05	0.62^**^	0.35	0.39^**^	0.10
Math performance	−0.16	0.02	−0.17^*^	0.02	−0.12	0.01	−0.16	0.02	−0.19^*^	0.03	−0.20^**^	0.03
Math confidence			−0.32^**^	0.04			−0.02	< 0.01			−0.27^*^	0.03
Math interest			−0.06	< 0.01			−0.29^**^	0.04			−0.07	< 0.01
Math importance			−0.27^**^	0.04			−0.38^**^	0.08			−0.23^**^	0.03
R^2^ change				0.24^**^				0.29^**^				0.20^**^
R^2^		0.42^**^		0.67^**^		0.28^**^		0.57^**^		0.49^**^		0.69^**^

* p < 0.05;

** *p < 0.01; n = 79*.

In the analyses predicting numerical confidence, general anxiety was again a consistent predictor, however it was a much weaker predictor in first grade, and was nonsignificant when attitudes were included. Math performance was consistently not a significant predictor across grades. For the math attitudes, different attitudes were important in different grades. Math interest was significant at first and third grades, math importance was significant at second and third grades, and math confidence was not significant at any grade. Similar to results for negative reactions and worry, we were better able to predict numerical confidence for older students (*R*^2^ = 0.30 at first grade, 0.50 at second grade, and 0.57 at third grade).

## Discussion

The goal of this study was to examine the reliability and validity of the MASYC as well as to examine the reliability and validity of a revised version of this measure (MASYC-R). We also examined how well we could predict math anxiety from its correlates. Our results suggest that we were able to develop an adapted version that avoids a number of issues that arose in the original version. Our final version of the scale includes eight of the original 12 items from the MASYC and five new items that we developed based on other scales. The majority of first and second grade students who participated in cognitive interviews interpreted all of the MASYC-R items appropriately. Therefore, we are confident that the scale can lead to valid inferences about math anxiety for children at this age. In addition, the subscales were correlated with general anxiety, other math attitudes, math performance, and gender as would be expected, providing some initial evidence for convergent validity.

### Reliability and factor structure

We have presented evidence for the reliability and construct validity of this new measure based on the results of CFAs with our revised scale. We removed items that were deemed to have poor construct validity. We were able to create a 13-item scale that had good reliability estimates and supported a three-factor structure similar, but not identical to, the original MASYC. We do want to point out that the initial MASYC scale was reasonably reliable in our sample. However, we were able to improve the reliability and also ensure that each of the items included was also well understood by young children (i.e., led to valid responses).

One thing that we noticed in our data is that our factors were much more highly correlated with each other than Harari et al. (2013) had initially found. We found this to be the case both in the test of the initial MASYC model and in our newly created version. Latent correlations between negative reactions and worry were around 0.9 (compared to 0.35 in Harari et al.) and correlations with numerical confidence were around 0.6, in comparison to 0.02 for negative reactions and 0.16 for worry reported by Harari et al. Because of the especially high correlation between negative reactions and worry, we estimated a model that combined these into one factor, but found that this combined model was a statistically poorer fit than when they are separate. This large correlation suggests these two factors are not as separable at this young age when compared with older populations, however; they are statistically not identical as they each have a significant amount of unique variance (Liebert and Morris, 1967).

### Levels of math anxiety

When examining whether there were students reporting high levels of math anxiety at this age, we found that a substantial subsample of children are already reporting elevated levels of math anxiety. Other studies show mixed results, with Ramirez et al. (2013) and Jameson (2013) reporting large proportions of students above the midpoint in math anxiety, whereas Wu et al. (2012) and Harari et al. (2013) report scale means that were substantially lower than the midpoint of their scales. Our results fit somewhere in the middle of these past studies and suggest that we can likely identify a small portion of children at this age who are at-risk for developing high levels of math anxiety.

### Validity

Cognitive interviews helped us assess if the scale items were understood as intended by young children. By eliminating items that were often answered based on inaccurate interpretations, we believe that our adapted version of the MASYC is more likely to lead to valid inferences about the math anxiety of young children. Overall, the results of these cognitive interviews helped us to think about ways to better assess math anxiety because students were often interpreting the questions in a much more literal fashion than we had initially anticipated. For example, the most frequent item to be misinterpreted, asked students if it would be fun to figure out how much money one needs to buy cookies and a drink. This led most students to answer based on how desirable it would be to purchase cookies or a drink, and not about how desirable it would be to do the math need to figure out how much money was required. They did not get the idea of what we were really asking about when we asked them less directly, and this was especially true for first-grade students.

Results supported previous evidence with older children and adults that math anxiety is significantly related to other attitudes about math including math confidence, math interest, and math importance (Hembree, 1990; Meece et al., 1990; Gierl and Bisanz, 1995; Krinzinger et al., 2009; Luo et al., 2009; Ahmed et al., 2012; Harari et al., 2013; Jameson, 2014; Graham and Morales-Chicas, 2015). The correlations that we found for math anxiety and confidence (~ −0.5 to −0.6) suggest that, although math anxiety and math confidence are often conceptualized as opposites, they may just be highly related constructs, though with measurement error, it is difficult to determine what led to correlations of this size. These correlations are similar to what Harari et al. (2013) reported when examining correlations between math anxiety and mathematics attitude, which was a combination of math confidence and interest (*r* = −0.62).

Our results showed that the negative reactions and worry factors were significantly correlated with math performance (−0.19 < *r*s < −0.20). These results suggest that at this age, math anxiety is weakly related to performance. Importantly we also found that, for the MASYC-R, these relations persisted after accounting for general anxiety (−0.13 < *r*s < −0.15), which was not the case for the original MASYC factors. The correlations that we found with math performance are on the lower range of those found by Harari et al. (2013; −0.15 < *r*s < −0.35) and on the lower range of those found by other researchers at this age (−0.19 < *r*s < −0.61; Gierl and Bisanz, 1995; Wu et al., 2012, 2014; Harari et al., 2013; Jameson, 2013; Ramirez et al., 2013, 2016; Vukovic et al., 2013; Cargnelutti et al., 2016). Interestingly, our results are quite different from those found by Harari et al. in their original paper. In that paper, they found that negative reactions and numerical confidence were related to math performance, but worry was not, whereas here we found no relation with numerical confidence and a significant relation with worry instead. It is unclear why we might have found such different results. However, there are some important differences between our samples that may help us to reconcile some of our differing results. Namely, their sample included ~100 first grade students, many of whom did not speak English as a first language. It seems reasonable that this could have contributed to differences in how students from our sample and their sample responded to items.

We found mixed results in regard to whether or not there were gender differences in math anxiety. We found a gender difference, but only in our newly adapted negative reactions and worry scales. Even though these factors did show a gender difference, the differences were no longer statistically significant once we accounted for the small gender difference in general anxiety. This demonstrates how important it is to measure general anxiety when examining math anxiety, especially when looking at relations with math performance or gender differences. These results correspond with much of the evidence that there are small or nonexistent gender differences in anxiety for children this age (Gierl and Bisanz, 1995; Harari et al., 2013; Ramirez et al., 2013; Jameson, 2014; Hill et al., 2016).

### Predicting math anxiety

We also were interested in how well we could explain math anxiety by using the other correlates in our study together in one model to gain understanding of the most critical predictors at this age. We were able to explain 50% of the variance in negative reactions, 41% in numerical confidence, and 53% in worry using general anxiety, math performance, and math attitudes (confidence, interest, and importance). All of the predictors except math performance were statistically significant in predicting numerical confidence. For negative reactions and worry, all of the predictors were significant with the exception of math importance (Gierl and Bisanz, 1995). General anxiety was, by far, the strongest predictor for negative reactions and worry, explaining 13 and 16% of the unique variance, respectively. For numerical confidence, however, general anxiety was similar in strength to math interest and importance, each explaining 3–4% of the variance. These results suggest that numerical confidence is operating differently from the other two math anxiety factors, as it has a weaker relation with both math performance and general anxiety, which would seem the most likely to be predictors of math anxiety, and a stronger relation with the math attitude variables.

When we examine these relations at the different grade levels, the results are consistent for some variables (i.e., general anxiety, math performance, to an extent), but not others (i.e., math confidence, interest, and importance). We should exercise caution in interpreting these results, however, as they include small sample sizes. It does appear that general anxiety is a very consistent predictor of math anxiety across grades; however, math performance is only a predictor of negative reactions and worry in the third grade. Particular attitudes seemed to be more related to math anxiety at different grades, with math interest being the only attitude predictor in first grade, very little predictive value of attitudes at second grade (with the exception of math importance for numerical confidence) and with all three attitudes as predictors in the third grade (though not in all models). In addition, we are able to explain more of the variance in each of the math anxiety factors in the older children. This is likely partially because measurement error decreases as students get older (Wigfield and Karpathian, 1991), but it is also likely that these variables become more related as students get older. The results suggest that the relations between math anxiety and other factors does differ at different grade levels, thus studying these relations over development could be a fruitful avenue for future research. More research with larger samples within each grade or longitudinal data is needed to further test these patterns of relations.

### Limitations and future directions

There are some limitations to the current study that point to directions for future research. First, the math assessment was researcher-developed and therefore has limited reliability and validity evidence. The test was fortunately found to have strong internal consistency in this sample, but more work should be done examining how these math anxiety factors are related to math performance using assessments with more extensive reliability and validity evidence.

In the present study, the youngest children involved were first grade students at the end of the school year. Thus, we do not know how this scale may perform with younger children. It is critical that future research examines whether these items make sense to students earlier in the first grade school year or in kindergarten, to see if this measure is valid and reliable in a younger age group.

A number of items on the scale were reversed coded to create a numerical confidence scale, which could potentially be problematic. The fact that these items loaded onto a separate factor could be because they were all reverse-coded, not necessarily because the content they asked about was more related to each other than to other items. Our results here show that this scale is not correlated with our measure of math performance. It is also important to point out that four of these items use the word “like” and one uses “fun,” making it unclear whether they are capturing the opposite of math anxiety, or numerical confidence as it is titled, or if it is really also capturing a general attitude about math or interest in mathematics. The results of the regression analyses suggest that it is more related to math attitudes than general anxiety and math performance when compared to the other math anxiety factors. It would be interesting to give adapted versions of these items that ask about anxiety in the same situations (e.g., doing math problems on the board in front of the class). We did not do that in the current study because we wanted to administer all of the original MASYC items. But, one could rephrase these items, for example, adapting the item about liking raising your hand to *Raising my hand in math class makes me nervous*. In doing so, we might improve the reliability of the negative reactions and worry factors as the internal consistency estimates could still be improved by potentially having more items in these factors. It is also possible that a separate factor about social math anxiety or math evaluation anxiety would emerge, similar to other work with children (Gierl and Bisanz, 1995; Wu et al., 2012; Jameson, 2013). It would also be interesting to develop additional items that can help us get at the physiological aspect of anxiety or that measure math evaluation anxiety specifically.

It is also important to note that all measures were collected within a very small time frame, and therefore these are concurrent data and no causal conclusions can be drawn based on the relationships uncovered in this work. Future research using experimental and/or longitudinal designs can help tease apart any causal relations between these variables.

## Conclusion

In this study we developed an adapted version of the MASYC, the MASYC-R, in which we removed items that were shown to be confusing to young children, and showed that this adapted version is reliable and valid in our sample. We hope that researchers examining math anxiety in young children will consider using this measure in their research. We have kept this measure to a reasonable length and it is appropriate for group administration, like the original version. Having reliable and valid available measures designed to assess math anxiety in young children is important because research, including the results of this study, suggests that we begin to see elevated levels of math anxiety in some students at this age and that it has numerous negative consequences for later mathematical development.

## Author contributions

Both CG and AM meet the four criteria for authorship. Both authors were involved in conceptualizing the study, collecting and analyzing the data, writing the manuscript, final approval of the submitted version, and both agree to be accountable for all aspects of the work.

### Conflict of interest statement

The authors declare that the research was conducted in the absence of any commercial or financial relationships that could be construed as a potential conflict of interest.

## Footnotes

^1^We also went back to the Harari et al. (2013) model and tested the item on the negative reactions scale and found that it fit better with negative reactions than with worry for these models as well. To preserve the original structure of Harari et al. scale, we did not report this model.

^2^We cannot test if this model has significantly worse fit than the previous model with 17 items because nonnested models cannot be compared when using WSLMV estimation.

^3^Correlations with math performance are conducted while taking grade into account because there are large grade differences in math performance.
