# Supplementary material for: The Development and Validation of a Revised Version of the Math Anxiety Scale for Young Children
Source: Front Psychol. 2016 Aug 24;7:1181. doi: 10.3389/fpsyg.2016.01181 (PMC4995220; doi:10.3389/fpsyg.2016.01181)
Supplement: Supplementary file 1 [file Table1.docx]

Supplementary Online Materials for:

The Development and Validation of a Revised Version of the Math Anxiety Scale for Young Children

Colleen M. Ganley and Amanda L. McGraw

Table S1. Summary of Results from Cognitive Interviews

| Item | Interpretation | Freq. | Example(s) |
| --- | --- | --- | --- |
| *MASYC Items* |  |  |  |
| 1) Math gives me a stomachache. | Intended | 8 | - “No I never got a stomachache during math.” |
|  | Unintended | 1 | - “No. It sounds silly.” |
| 2) When it is time for math my head hurts. | Intended | 8 | - “No. Math doesn't make my head hurt.” |
|  | Unintended | 1 | - “Sometimes it hurts just because it aches.” |
| 3) When it is time for math my heart beats fast. | Intended | 4 | - Researcher: No? So what did you think we meant by your heart beats fast?  Child: Like you're so nervous that you can't even breathe. |
|  | Unintended | 5 | - [after showing no math anxiety on other items and saying loved math] Child: My heart always beats fast when it's time for math.  Researcher: Are you excited or nervous?  Child: Excited.  - “Yes because we usually have recess before math.”  - “Because I've been using a lot of exercise before I do math, so because we do playground right before we do math.” |
| 4) Figuring out if I have enough money to buy cookies and a drink is fun.* | Intended | 1 | - “I kind of like counting money.” |
|  | Unintended | 8 | - “I don't like cookies as much ‘cause they're not healthy for you.”  - “Because I really don't like milk.”  - “I like cookies and a drink.” |
| 5) I like doing math problems on the board in front of the class.* | Intended | 8 | - “I picked that one because I get to show them what I learned and what they need to learn.” |
|  | Unintended | 1 | - “Because I like to use the smartboard.” |
| 6) I like to raise my hand in math class.* | Intended | 8 | - “I don't really like doing it on, I don't really like raising my hand because sometimes it’s hard.” |
|  | Unintended | 1 | - “Well I kinda like it because my arm gets tired.” |
| 7) I like doing a math problem like this: 124 + 329* | Intended | 9 | - “Yes, because it’s pretty hard”  - “Because those are really really big numbers. I haven't even learned that in school yet.” |

| Item | Interpretation | Freq. | Example(s) |
| --- | --- | --- | --- |
| 8) I like being called on in math class.* | Intended | 8 | - “Because I like being called on in math class so I can learn something new everyday.” |
|  | Unintended | 1 | - “I like to use the smart board.” |
| 9) I get nervous about making a mistake in math. | Intended | 8 | - “Because I learn when I make mistakes.” |
|  | Unintended | 1 | “Because I'm usually the one that gets everything right.” (coded unintended because more general than math and more about perfectionism) |
| 10) When the teacher calls on me to tell my answer to the class, I get nervous. | Intended | 9 | - “I hardly get nervous when I tell my answer to the class”  - “Because it’s kinda a risk going up to the smart board” |
| 11) I am scared in math class. | Intended | 7 | - “Because I'm not scared”  - “A lot of times when I'm about to do my fluency I get really skittish.” |
|  | Unintended | 2 | - “Cause first grade, we don't have math class, we just teach in our own class.”  - “Because even though they have to turn some lights off, there’s still a row of lights on. So I'm not really scared.” |
| 12) Getting out my math books makes me nervous. | Intended | 4 | - “I'm like why did they ask that question. I'm never nervous to take out my math books.” |
|  | Unintended | 5 | - “I don't have a math book.”  - “No because our teacher hands it out.” |
|  |  |  |  |
| *Newly-developed Items* |  |  |  |
| 13) When it’s time for math I get butterflies in my stomach. (researcher-developed)  (dropped after 6 students) | Intended | 3 | - Child: I know what that means but…  Researcher: You don't get butterflies in your stomach before math?  Child: Nu-uh. |
|  | Unintended | 3 | - “Researcher: Have you had that feeling before?” Child: No (2 children)  - “That means when you have like um it’s another word of saying my stomach is grouchy, he’s really hungry.” |

| Item | Interpretation | Freq. | Example(s) |
| --- | --- | --- | --- |
| 14) I get worried before I take a math test. (Ramirez et al., 2013; Wu et al., 2012) | Intended | 9 | - “Not really. I usually feel cool, but sometimes I think maybe this math test is going to be hard because the unit's hard but usually it’s pretty easy in the end.” |
| 15) I feel nervous when the teacher is showing the class how to solve a math problem. (Jameson, 2013)  (dropped after 6 students because similar to #17) | Intended | 6 | - “Not really, because sometimes I don't really get it.” |
| 16) My heart starts to beat fast if I have to do math in my head. (Gierl & Bisanz, 1995; Harari et al., 2013) | Intended | 9 | - “No. I can really do math in my head pretty easy.”  - “Sometimes if it’s like big numbers.” |
| 17) I get nervous when my teacher is about to teach something new in math. (Wu et al., 2012) | Intended | 9 | - Child: “I would kind of get nervous.”  Researcher: “Why do you get a little nervous?”  Child: “Because I might not understand it.”  - “Because I like when my teacher teaches us something new.” |
| 18) I get worried when I don't understand something in math. (Wu et al., 2012) | Intended | 9 | - “Well if I don't understand it I feel like now there's a lot more to do, doing the whole subject thing and so I feel oh no if I fail this, I'm gonna fail everything.”  - “No, because I don't get worried a lot. I only do it when I need it in math test.” |
| 19) I get nervous when I see a page of math problems that I need to solve. (Suinn, Taylor, & Edwards, 1988)  (dropped after 6 students all said “No”) | Intended | 6 | - “Because I can, I’m really smart and I can do the math pages in a few seconds.”  - “I don't get nervous.” |
| 20) I feel nervous when I think about a math test I have the next day. (Chiu & Henry, 1990)  (dropped after 6 students because similar to #14) | Intended | 6 | - “Because I really like math tests.”  - “I picked no because I actually am not afraid of math tests, they're just really easy.” |

| Item | Interpretation | Freq. | Example(s) |
| --- | --- | --- | --- |
| 21) I feel nervous when I am doing math. (researcher-developed)  (added after 6 students) | Intended | 3 | - “Nope.” |

*Note.* Freq. = Frequency of response. Light gray items are those that were excluded before the main survey administration. Medium gray items are those for which a number of students did not interpret the items as intended and they were later dropped. The dark gray item was later excluded for theoretical/empirical reasons. Citations in parentheses indicate what scale a new item was adapted from.
